# Description of *Longidorella (Saevadorella) caspica* n. sp. (Dorylaimida: Nordiidae) from north Iran

**DOI:** 10.21307/jofnem-2021-004

**Published:** 2021-02-15

**Authors:** Fariba Heydari, Mohammad Reza Atighi, Ebrahim Pourjam, Majid Pedram

**Affiliations:** Department of Plant Pathology, Faculty of Agriculture, Tarbiat Modares University, Tehran, Iran

**Keywords:** 28S rDNA D2-D3, Molecular, Morphology, New species, Phylogeny, Taxonomy

## Abstract

This contribution provides the morphological and molecular identification of a new species of the genus *Longidorella* (*Saevadorella*). *L. (S.) caspica* n. sp., was recovered from the rhizospheric soil of grasses in Mazandaran province in the seashore of the Caspian Sea. It is characterized by females with a length of 788 to 874 μm and a cephalic region with prominent papillae; and separated from the rest of the body by a remarkable constriction, an odontostyle of 32 to 33 μm, vulva at 52.5 to 59.0%, and a tail of 33 to 38 μm with a rounded tip. Males have 32 to 35 μm long spicules of dorylaimoid form and five to seven ventral supplements ending at 48 to 55 μm distance from cloacal pair. The new species was morphologically compared with seven nominal species viz. *L. (S.) arenicola, L. (S.) cuspidata, L. (S.) magna, L. (S.) perplexa, L. (S.) saadi*, *L.* (*S*.) *saevae*, and *L. (S.) tharensis*. It was further compared with similar species under the subgenus *Longidorella (Enchodorella)* viz. *L. (E.) deliblatica* and *L. (E.) murithi.* Besides morphological studies, molecular phylogenetic studies using partial sequences of D2 to D3 expansion fragments of 28S rDNA were performed for the new species and its phylogenetic relationships with other species and genera were discussed.

Andrássy (2009) presented a taxonomic history of the family Nordiidae (Jairajpuri and Siddiqi, 1964). According to him, the main characteristics of the family are a needle-like odontostyle with a fine aperture, often flanged odontophore and similar tail in sexes. The needle-like odontostyle probably makes the members capable to parasitize lower or higher plants (Peña-Santiago, 2021). Although some nordiids are recovered from rhizosphere of higher plants (e.g. *Longidorella (Actinolamoides) foveola* Siddiqi, 2007, *L. (Actinolamoides) ecuadorica* Siddiqi, 2007, and *L. (Longidorella) miniparva* Siddiqi, 2007), these nematodes have not been reported as economically important crop pests, and the studies dealing with their plant parasitism ability are poor.

The genus *Longidorella* (Thorne, 1939), currently, with 39 known species, belongs to the subfamily Nordiinae (Jairajpuri and Siddiqi, 1964), with *L. parva* (Thorne, 1939) as its type species. The small and stout body, large amphids, long needle-like odontostyle, and small pharyngeal bulb are characteristics of this genus (Andrássy, 2009). Siddiqi (2007) reviewed the taxonomy of the genus, listed its synonym genera, added several species, and categorized the species under seven subgenera. The subgenus *Saevadorella* (Siddiqi, 1982) is one of them, being characterized mostly by its offset cephalic region, amphidelphic female reproductive system, a usually transverse vulval slit and usually sclerotized vagina.

The history of taxonomic studies on nordiids in Iran is given by Heydari et al. (2020). During the present study, one population of a nordiid nematode was recovered from a sandy soil sample collected in seashore of Caspian Sea in Mazandaran province. Its preliminary studies revealed it belongs to *Longidorella*. The detailed comparisons revealed that the recovered species belongs to the subgenus *Saevadorella* based upon its morphological characters (see Discussion) and was described and illustrated herein as *L* (*S*.) *caspica* n. sp. Thus, the present study aims to describe and illustrate the new species, and unravel its phylogenetic relationships with other spices.

## Materials and methods

### **S**ampling, extracting, mounting, and drawing

Several soil samples were collected from north Iran close to Caspian Sea during 2014. The new species was recovered from soil of grasses, using the tray method (Whitehead and Hemming, 1965). They were handpicked under a Nikon SMZ1000 stereomicroscope, heat-killed by adding boiling 4% formalin solution, transferred to anhydrous glycerin according to De Grisse (1969), mounted on permanent slides, and examined using a Nikon Eclipse E600 light microscope. Photographs were taken using an Olympus DP72 digital camera attached to an Olympus BX51 microscope powered with differential interference contrast. Drawings were made using a drawing tube attached to the microscope and were redrawn using CorelDRAW software version 17. The location of pharyngeal glands’ nuclei was calculated following Andrássy (1998). The proposed taxonomic frame by Siddiqi (2007) as followed by Nasira et al. (2010) was followed in this study.

### **D**NA extraction, PCR, and sequencing

One live female nematode of the new species was used for DNA extraction. The specimen was washed and observed under a temporary slide, transferred to a small drop of TE buffer (10 mM Tris-Cl, 0.5 mM EDTA; pH 9.0) on a clean slide and squashed using a clean cover slip, and the pressure of a plastic pipette tip. The suspension was collected by adding 15 μl TE buffer. The DNA sample was stored at −20°C. Primers for 28S rDNA D2-D3 amplification/sequencing were forward primer D2A (5´-ACAAGTACCGTGAGGGAAAGT-3´) and reverse primer D3B (5´-TCGGAAGGAACCAGCTACTA-3´) (Nunn, 1992). The PCR cycles and sequencing of amplified fragments were according to Jahanshahi Afshar et al. (2019) and sequenced directly for both strands using the same primers with an ABI 3730XL sequencer (Bioneer Corporation, South Korea). The newly generated sequence for the new species was deposited in the GenBank database under the accession number MH346475.

### **P**hylogenetic analysis

The newly generated sequence in this study was compared with those of other relevant sequences from other nematodes deposited in the GenBank database using the BLAST homology search program. Several 28S rDNA D2-D3 sequences of nordiid and other dorylaimid taxa were downloaded. Two sequences of mononchid species were used as outgroups (for species names, accession numbers, and related plants, see Table 1). The sequences were aligned using the Q-INS-i algorithm of the online version of MAFFT version 7 (http://mafft.cbrc.jp/alignment/server/) (Katoh and Standley, 2013). The poorly aligned positions and divergent regions were eliminated using the online version of Gblocks 0.91b (Castresana, 2000) using all three less stringent options (Mobasseri et al., 2019; Panahandeh et al., 2018). The model of base substitution was selected using MrModeltest 2 (Nylander, 2004). The Akaike-supported model, a general time-reversible model including among-site rate heterogeneity and estimate of invariant sites (GTR + G + I), was selected for phylogenetic analysis. Bayesian analysis was performed using MrBayes v3.1.2 (Ronquist and Huelsenbeck, 2003), running the chains for two million generations. Burn-in phase was set at 25% of the converged runs. The Markov chain Monte Carlo (MCMC) method within a Bayesian framework was used to estimate the posterior probabilities of the phylogenetic tree (Larget and Simon, 1999) using the 50% majority rule. To visualize the results of each run in order to check the effective sample size of each parameter, Tracer v1.5 (Rambaut and Drummond, 2009) was used. The output file of MrBayes was visualized using Dendroscope v3.2.8 (Huson and Scornavacca, 2012) and was drawn in CorelDRAW version 17.

**Table 1.** The used sequences in 28S phylogeny of *Longidorella (Saevadorella) caspica* n. sp., their accession number, locality, and related plants.

Species name	Accession number	Location, related plant
*Allodorylaimus* sp.	KY942069	China, unknown
*Allodorylaimus andrassyi*	AY593016	Unknown
*Allodorylaimus andrassyi*	AY593015	Unknown
*Dorydorella bryophila*	HM235514	Unknown
*Enchodelus* sp.	KP190120	Hamedan province, Iran, mosses
*Enchodelus longispiculus*	KP190119	Hamedan province, Iran, mosses
*Enchodelus* sp.	EF207240	Unknown
*Enchodorus dolichurus*	KR184125	Golestan province, Iran, mosses
*Enchodorus dolichurus*	KR184124	Golestan province, Iran, mosses
*Enchodorus yeatsi*	KX691911	East Azarbaijan province, Iran, grasses
*Eudorylaimus* sp.	AY593037	Unknown
*Heterodorus brevidentatus*	KP963962	Kerman city, Iran, mosses
*Heterodorus brevidentatus*	KP963960	Maragheh city, Iran, mosses
*Heterodorus brevidentatus*	KP963963	Maragheh city, Iran, mosses
*Heterodorus brevidentatus*	KP963964	Kermanshah city, Iran, mosses
*Heterodorus brevidentatus*	KP963965	Kermanshah city, Iran, mosses
*Heterodorus brevidentatus*	KP963961	Tehran city, Iran, mosses
*Heterodorus morgensis*	KX691912	Mazandaran province, Iran, grasses
*Heterodorus youbertghostai*	KR184126	Sabalan region, Iran, grasses
*Heterodorus youbertghostai*	KR184127	Sabalan region, Iran, grasses
*Longidorella macramphis*	AY593042	Unknown
*Longidorella* sp.	AY593043	Unknown
*Longidorella penetrans*	HM235515	Unknown
*Longidorella* sp.	AY593044	Unknown
*Longidorella* sp.	AY593045	Unknown
*Mesodorylaimus* sp.	AY593006	Unknown
*Mesodorylaimus* sp.	AY593005	Unknown
*Microdorylaimus miser*	AY593046	Unknown
*Microdorylaimus modestus*	HM235513	Unknown
*Microdorylaimus modestus*	AY593049	Unknown
*Mononchus truncatus*	AY593064	Unknown
Nordiidae sp.	KP202362	Tehran city, Iran, grasses
Nordiidae sp.	KP202361	Tehran city, Iran, grasses
Nordiidae sp.	AY593054	Unknown
Nordiidae sp.	MH346478	Tehran city, Iran, mosses/pine tree
*Prionchulus punctatus*	MG994945	Unknown
*Prodorylaimus uliginosus*	AY593034	Unknown
*Pungentus engadinensis*	MH346473	Semnan province, Iran, fruit trees
*Pungentus engadinensis*	MH346474	Mazandaran province, Iran, forest trees
*Pungentus silvestris*	AY593052	Unknown
*Pungentus silvestris*	AY593054	Unknown
*Pungentus silvestris*	AY593053	Unknown
*Pungentus azarbaijanensis*	MH346476	West Azarbaijan province, Iran, grasses
*Pungentus azarbaijanensis*	MH346477	West Azarbaijan province, Iran, grasses
*Pungentus engadinensis*	AY593050	Unknown
*Pungentus monohystera*	MF325344	Germany, lime tree
*Pungentus monohystera*	MF325343	Germany, lime tree
*Rhyssocolpus vinciguerrae*	KP204547	Gilan province, Iran, forest trees
*Thonus minutus*	AY593047	Unknown
*Thonus minutus*	AY593048	Unknown
*Thonus circulifer*	AY593039	Unknown
*Thonus* sp.	AY593041	Unknown
*Thonus* sp.	AY593040	Unknown
*Thonus circulifer*	AY593038	Unknown
*Tylencholaimellus* sp.	AY593055	Unknown

## Results

### Longidorella (Saevadorella) caspica n. sp.

(Figs. 1-3).

**Figure 1:**
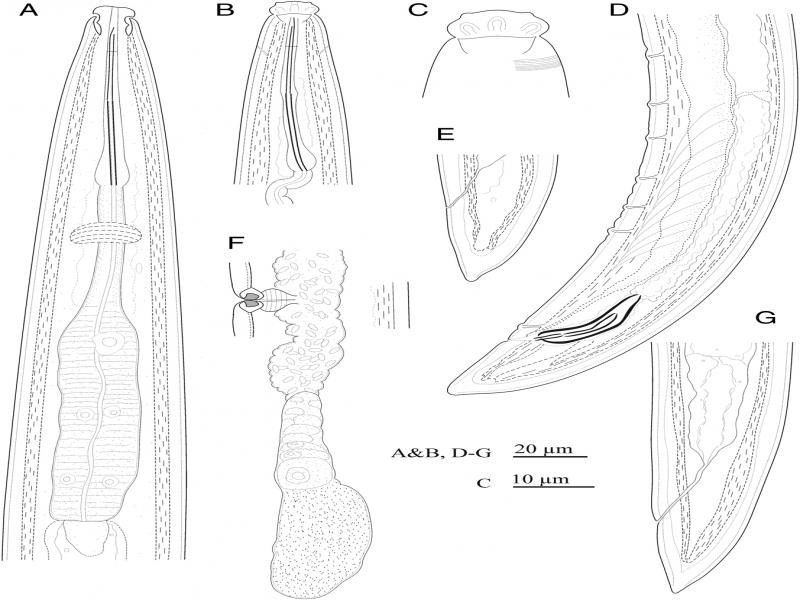
Line drawings of *Longidorella (Saevadorella) caspica* n. sp. A-C, E-G: Female; D: Male. A: Pharynx; B: Anterior body region; C: Anterior end; D: Posterior body region; E and G: Tail and posterior body region, F: Posterior genital tract.

**Figure 2:**
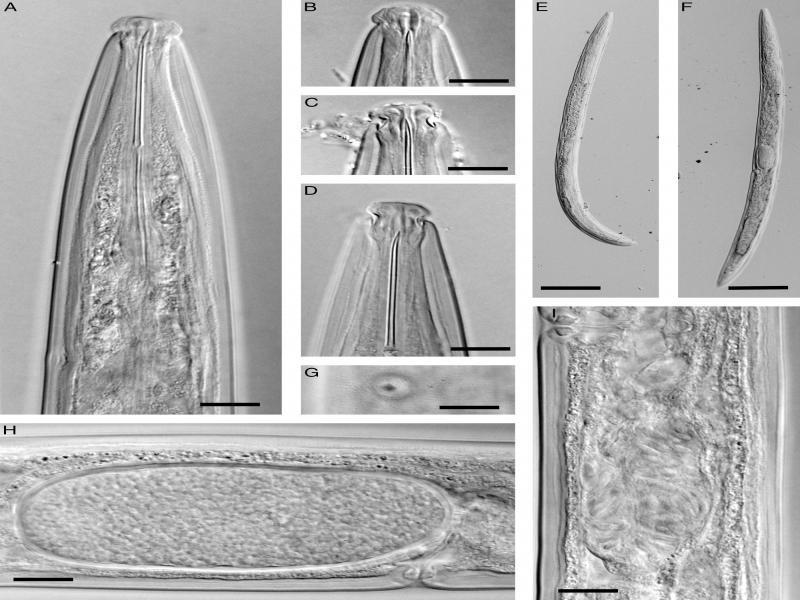
Photomicrographs of *Longidorella (Saevadorella) caspica* n. sp. A-D, F-I: Female; E: Male. A-D: Anterior and cephalic region; E and F: Entire body; G: Vulva in ventral view; H: Mature egg inside female reproductive system; I: Sperm inside the uterus. (Scale bars = 10 μm, except E and F = 100 μm).

**Figure 3:**
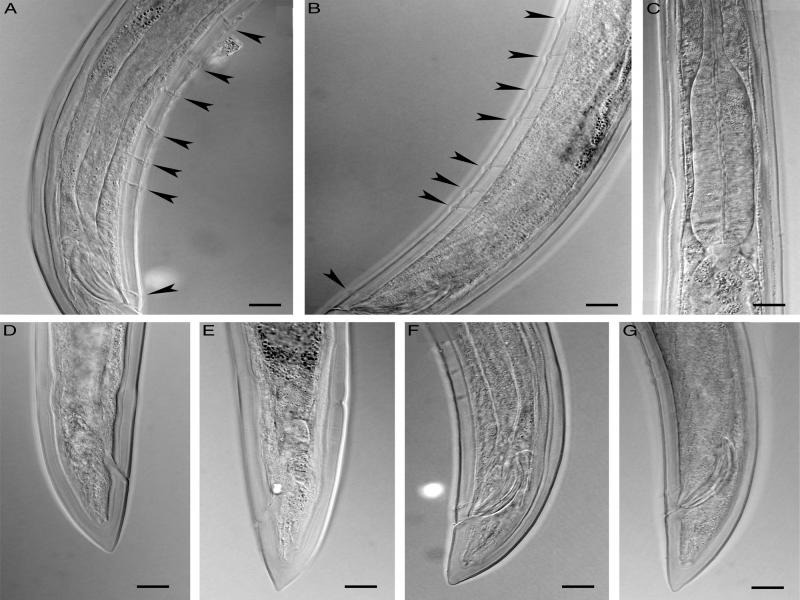
Photomicrographs of *Longidorella (Saevadorella) caspica* n. sp. A and B, F and G: Male; C-E: Female. A and B: Posterior body region (Arrows showing supplements). C. Pharyngeal bulb. F-G: Posterior body region with spicules. (All scale bars = 10 μm).

### Measurements

Measurements of the new species are given in Table 2.

**Table 2.** Morphometrics of *Longidorella (Saevadorella) caspica* n. sp.

	Holotype	Paratype	
Characters	Female	Females	Males
*n*	1	3	3
*L*	852	832 ± 38.5 (788-874)	811 ± 74 (725-856)
*a*	19.8	19 ± 2 (16.8-21.3)	22.0 ± 1.5 (20.7-23.8)
*b*	3.8	3.7 ± 0.2 (3.5-3.8)	3.8 ± 0.2 (3.6-4.0)
*c*	25.8	27.2 ± 7 (20.7-37.0)	25.2 ± 2.2 (23.0-27.5)
*c′*	1.3	1.3 ± 0.3 (0.9-1.5)	1.3 ± 0.1 (1.2-1.4)
*V*	59.2	55.7 ± 3.0 (52.5-59.0)	–
Anterior end-vulva	504	463.5 ± 32.5 (425-504)	–
Cephalic region diam.	12	11.5 ± 1.0 (10-12.5)	12.5 ± 0.5 (12-13)
Cephalic region height	5	5.0 ± 0.0 (5-5)	4.7 ± 0.6 (4-5)
Odontostyle length	33	33.0 ± 0.5 (32-33)	35 ± 1 (34-36)
Odontophore length	38	35.5 ± 2.5 (33-38)	33.3 ± 3.0 (30-36)
Stylet total length	71	68.5 ± 2.0 (66-71)	68.5 ± 2.5 (66-71)
Guiding ring from ant. end	2.8	18 ± 1 (18-20)	18.0 ± 0.5 (18.0-18.5)
Neck length	18	229 ± 2 (227-231)	212 ± 8 (203-220)
Pharyngeal expansion length	92	93 ± 1 (92-94)	85.5 ± 5.5 (80-91)
Diam. at guiding ring level	16	18.5 ± 0.0 (18.5-18.5)	18.5 ± 0.0 (18.5-18.5)
-at neck base	40	41.0 ± 3.5 (38-46)	36.5 ± 2.5 (34-39)
-at mid-body	43	44.0 ± 2.5 (41-47)	37.0 ± 2.5 (35-40)
-at anus	25	25.0 ± 0.5 (24-25)	25 ± 1 (24-26)
Prerectum	56	57.0 ± 7.5 (47-65)	81 ± 20 (67-95)
Rectum	22	23.5 ± 3.5 (19-27)	38.0 ± 1.5 (37-39)
Tail length	33	34.5 ± 2.5 (33-38)	32.5 ± 4.0 (29-37)
Spicules length	–	–	33.0 ± 1.5 (32-35)

**Note:** All measurements are in μm and in the form Mean ± SD (range).

### **D**escription

#### 

Female: Body fusiform, tapering gradually towards both ends, very slightly curved ventrad. Cuticle with two layers, 3 to 4 μm thick at anterior body region and mid body, 4 to 5 μm thick at the anterior lip of anus, with very delicate transverse striae visible at the dorsal side of the tail. Cephalic region separated from the rest of the body by a deep constriction. Labial papillae protruding, large and distinct. Amphidial fovea cup-shaped, large, their opening ca. 75% of cephalic region width wide, at the level of constriction. Odontostyle long and thin, 2.5 to 3.0 times longer than cephalic region width. Odontophore rod-like, its base simple, muscles at its base slightly swollen, but not flanged or sclerotized, approximately equal in size with odontostyle or slightly longer. Guiding ring single. Pharynx dorylaimoid, the anterior part narrower, enlarging gradually to the pharyngeal bulb. Location of pharyngeal glands’ nuclei follow: DN = 65 to 68, S1N = 32 to 40, S2N = 76 to 79. Cardia hemispheroid, 10-17 × 9-12 μm. Intestine simple with no specific features. Reproductive system didelphic-amphidelphic, genital branches 150 to 160 μm long, each branch composed of an ovary 90 to 120 μm long, oviduct and sphincter, a tubular uterus 55 to 60 μm long usually containing sperm, vagina 30 to 37% of body width, composed of *pars proximalis vaginae* 8-10 × 7-10 μm in size, *pars refringens vaginae* with two weakly sclerotized pieces triangular to trapezoid with 4 μm height and 3 to 5 μm width, *pars distalis vaginae* 2 to 4 μm long, and vulva a small rounded pore. Prerectum twice the anal body diameter and rectum equal to the anal body width in length. Tail conical with finely rounded tip, ventrally nearly straight, dorsally convex with a very weak dorsal concavity at the end, appearing slightly sub-digitate.

#### 

Male: Similar to females in general morphology except for the posterior body end more ventrally bent. Spicules dorylaimoid, almost slender, about five times longer than wide, their head (capitulum) narrow, a well-developed hump and deep hollow (*sensu* Peña-Santiago et al., 2014) lacking. Lateral guiding pieces cylindroid, 8 to 10 μm long. The copulatory supplements composed of a cloacal pair at 7 to 10 μm anterior to cloacal aperture, and a series of five to seven ventromedian supplements ending at 48 to 55 μm from the cloacal pair. Tail similar to that in female.

#### **T**ype habitat and locality

Rhizosphere of grasses, Mazandaran province, north Iran. GPS coordinates: 36°38ʹ6.225ʺN, 51°33ʹ52.236ʺE.

#### **T**ype specimens

Holotype female, paratype females and males were deposited in Nematology Collection of Faculty of Agriculture, Tarbiat Modares University, Tehran, Iran (slide accession codes: TM5100-TM5103). The ZooBank Life Science Identifier (LSID) for this publication is as follows: urn:lsid:zoobank.org:pub:1915B3CE-4B79-480B-8117-E926CA3CD090.

#### **E**tymology

The specific epithet was derived from Caspian Sea, from where the new species was recovered in its vicinity.

#### **D**iagnosis and relationships

*Longidorella* (*Saevadorella*) *caspica* n. sp. is mainly characterized by its cephalic region separated from the rest body by a deep constriction and prominent labial papillae. It was further characterized by 788 to 874 μm long females having 92 to 94 μm long odontostyle in females and 80 to 91 μm in males, with didelphic-amphidelphic reproductive system, vulva at 52.5 to 59.0%, 33 to 38 µm long dorsally convex tail in females, males with 32 to 35 µm long dorylaimoid spicules, and five to seven ventral supplements.

The new species was morphologically compared with seven nominal species under the subgenus *viz*. *Longidorella* (*Saevadorella*) *arenicola* (Vinciguerra and Zullini, 1980), *L.* (*S.*) *cuspidata* (Andrássy, 1964; Jairajpuri and Hooper, 1969), *L. (S.) magna* (Loof, 1971), *L. (S.) perplexa* (Siddiqi, 2007), *L. (S.) saadi* (Siddiqi, 2007), *L. (S.) saeva* (Siddiqi, 1982) and *L. (S.) tharensis* (Nasira et al., 2010) as follow.

From *Longidorella (Saevadorella*) *arenicola* by a longer body of female (788-874 vs 630-750 μm), shorter odontostyle (32-33 vs 40-42 μm), greater b (3.5-3.8 vs 2.7-3.3) value, pore-like vulval opening (vs transverse slit), less ventromedian male copulatory supplements (five to seven vs nine) with the last supplement distantly placed to the spicules’ head (vs close).

From *L.* (*S.*) *cuspidata* by a longer body of female (788-874 vs 510-760 μm), greater a (16.8-21.3 vs 12.5-14.7), b (3.5-3.8 vs 2.6-3.0), and c (20.7-37.0 vs 16.5-20.0) ratios, vulva pore-like (vs transverse, after its original drawing) and tail ventrally slightly convex (vs concave).

From *L. (S.) magna* by a shorter body of female (788-874 vs 1,050-1,100 μm), longer odontophore (33-38 vs 20-24 μm) and shorter tail (33-38 vs 47-50 μm).

From *L. (S.) perplexa* by a longer body of female (788-874 vs 580-680 μm), greater b (3.5-3.8 vs 2.5-3.0), anteriorly located vulva (V = 52-59 vs 58-67), shorter odontostyle (32-33 vs 38-46 μm), shorter odontophore (33-38 vs 39-44 μm), pore-like vulval opening (vs transverse slit), longer spicules (32-35 vs 31-32 μm), and five to seven male copulatory supplements (vs three).

From *L. (S.) saadi* by a shorter odontostyle (32-33 vs 40-50 μm), shorter odontophore (33-38 vs 40-44 μm), less c′ (0.9-1.5 vs 1.4-1.7) value, different range of V (52.5-59.0 vs 54-66), longer prerectum (47-65 vs 20-30 μm) and shorter spicules (32-35 vs 40 μm).

From *L. (S.) saeva* by shorter odontostyle (32-33 vs 39-42 μm), shorter odontophore (33-38 vs 42-48 μm), pore-like vulval opening (vs transverse), smaller c′ (0.9-1.5 vs 1.2-2.0), shorter spicules (32-35 vs 38-40 μm) and number of copulatory supplements of male (five to seven vs two).

From *L. (S.) tharensis* by cephalic region separated from the rest body by a deep constriction (vs slightly offset), shorter odontostyle (32-33 vs 35-38 μm), longer neck (227-231 vs 197-223 μm), longer pharyngeal bulb (92-94 vs 67-68 μm), pore-like vs transverse vulval slit and longer tail of female (33-38 vs 20.2-26.4 μm).

Based on similar general morphology, the new species was further compared with two species under the subgenus *Longidorella (Enchodorella)* (Siddiqi, 2007) viz. *L. (E.) deliblatica* (Krnjaic, 1971) and *L. (E.) murithi* (Altherr, 1950) as follow.

From *L. (E.) deliblatica* by a longer body (788-874 vs 602-700 μm), slightly longer odontostyle (32-33 vs 26.8-31.4 μm) and odontophore (33-38 vs 22.4-30.0 μm), narrower tail tip (vs wider) and almost slender spiclues (vs with deep hollow).

From *L. (E.) murithi* by a cephalic region separated from the rest body by a sharp constriction (vs not), shorter odontostyle (32-33 vs 40-44 μm) and tail tip not remarkably narrowing (Andrássy, 2009).

### **M**olecular phylogenetic relationships

#### **D**2-D3 fragments of 28S rDNA phylogeny

**Figure 4:**
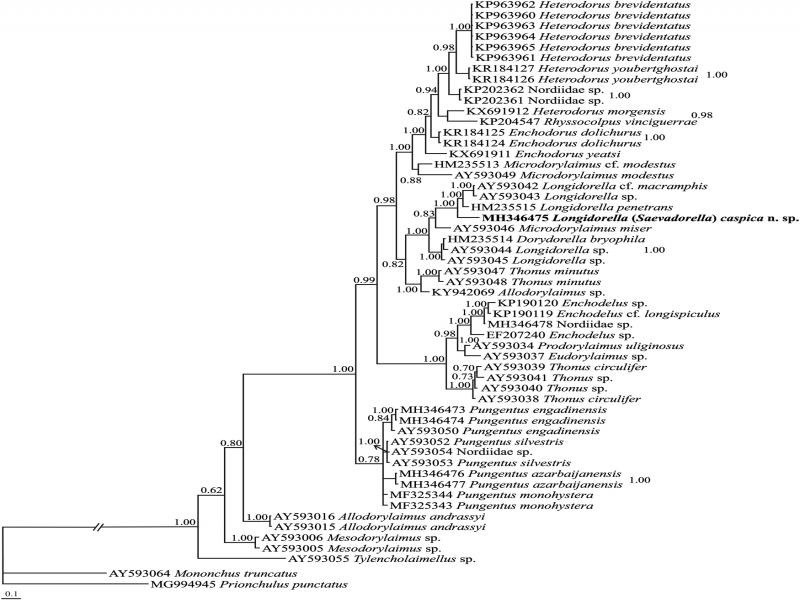
Bayesian 50% majority rule consensus tree inferred from the 28S rDNA D2-D3 sequences of *Longidorella (Saevadorella) caspica* n. sp. under the GTR + G + I model. Bayesian posterior probabilities (BPP) and maximum likelihood bootstrap (ML BS) values *>* 0.50 are given for appropriate clades in the form: BPP/ML BS. The newly generated species is in bold font.

Sequencing of D2-D3 expansion segments of 28S rDNA of the new species yielded a single fragment of 800 nt long. A total number of 53 sequences of 28S rDNA D2-D3 of *Longidorella* spp., other nordiids as well as some other dorylaimids (ingroup sequences) were retrieved from GenBank for phylogenetic analyses. The outgroup sequences belonged to two mononchid species (Table 1). Figure 4 represents the Bayesian phylogenetic tree reconstructed using this dataset. The nordiid genera occupied separate placements in this tree. The new species fell into a highly supported major clade including five other sequences of the genus *Longidorella* and two other dorylaimid genera. The clade including two sequences with accession numbers AY593042 and AY593042 assigned to *Longidorella* sp. and *Longidorella* cf. *macramphis*, represents the tentative sister clade to the new species.

## Discussion

In this study, one species of the genus *Longidorella* was recovered in north Iran. It was assigned to the subgenus *Saevadorella sensu* (Siddiqi, 2007) mainly by having offset cephalic region, sclerotized vagina, amphidelphic reproductive system of females and not twisted posterior body region; and was described and illustrated using morphological and molecular approaches. The genus has been divided into seven subgenera by Siddiqi (2007). Such an artificial grouping can be helpful for species identification under this specious genus, but, the qualitative traits delimiting the subgenera, sometimes fail to well distinguish them, and as the result, species under the similar subgenera need to be compared with all similar forms. Thus, the new species was compared with species under both subgenera *Longidorella* (*Saevadorella*) and *L*. (*Enchodorella*). In the proposed framework by Andrássy (2009), however, all species have unified under the genus *Longidorella*. Lacking of molecular data of type populations of most species of the genus or poor descriptions of the species and inaccessibility of the type specimens are the main obstacles in taxonomy of the genus.

Currently, GenBank database is poor for molecular data of nordiids, and based on the currently available ribosomal RNA sequences, the family is not monophyletic (present study; Pedram et al., 2011; Álvarez-Ortega, 2020; Peña-Santiago et al., 2015).

The newly described species in present study has been characterized using both traditional and molecular data, and further future molecular data will help better clarifying the phylogeny of the genus.
